# Surgery Utilization Mediates Survival in Patients With Dementia and Stage I–III Colorectal Cancer

**DOI:** 10.1111/jgs.70643

**Published:** 2026-08-07

**Authors:** Evan M. Abbey, Vashisht V. Madabhushi, Meghan E. Lark, Brian Ruedinger, Malaz A. Boustani, Sanjay Mohanty

**Affiliations:** ^1^ Surgical Outcomes and Quality Improvement Center Indiana University Indianapolis Indiana USA; ^2^ Department of Medicine Indiana University School of Medicine Indianapolis Indiana USA; ^3^ Center for Aging Research Indiana University Indianapolis Indiana USA

**Keywords:** colorectal cancer, dementia, surgery

## Abstract

**Background:**

Dementia is associated with increased mortality among patients with colorectal cancer (CRC), and they receive less invasive evaluations, chemotherapy, and surgery. The objectives of this study were to (1) compare demographics and treatment patterns among individuals with CRC with and without dementia, and (2) evaluate the relationship between dementia, surgery receipt or refusal, and survival.

**Methods:**

The Indiana Cancer Registry was reviewed (2000–2020) for adult patients with clinical Stage I–III CRC. The diagnosis of dementia was determined by the presence by ICD‐9/10 codes. Overall survival was estimated using the Kaplan–Meier method and compared between patients with and without dementia using the log‐rank test. To assess the extent to which the association between dementia and survival was mediated by surgery utilization, a regression‐based causal mediation analysis of surgery receipt and refusal was performed, using covariates chosen a priori considered possible confounders of diagnosis, treatment, and survival.

**Results:**

A total of 69,318 patients met the inclusion criteria; 1516 (2.1%) had dementia and 67,802 (97.8%) did not. The dementia cohort had a higher percentage of female patients (55% vs. 49%, *p* < 0.001) and was older, with a median age at diagnosis of 79 (vs. 69, *p* < 0.001). Patients without dementia had higher survival probabilities across all time points (12, 36, 60, 120, and 180 months), and the survival difference was statistically significant (*p* < 0.001). Receipt of surgery was a statistically significant mediator of survival in patients with dementia and CRC, with a proportion mediated of 9.6% (95% CI 6.2%–13.2%).

**Conclusion:**

Individuals with dementia and CRC receive lower rates of surgery with higher rates of refusal. Differences in surgical utilization are a significant mediator of survival differences, emphasizing the importance of patient‐centered goals‐of‐care discussions with patients and family members to assess the benefits and burdens of surgical treatment when planning.

## Summary

- Key Points
  ○ Patients with colorectal cancer and dementia had lower rates of surgery due to contraindications and refusal, compared to those with colorectal cancer without dementia.
  ○ The differing practice in surgery utilization between patients with and without dementia is a significant mediator of the survival differences between the two groups.
- Why does this paper matter?
  ○ This is the first study to quantify how differences in surgical utilization among individuals with dementia and colorectal cancer influence survival.
  ○ Patients with both conditions are less likely to receive surgery than those without dementia, and this difference significantly mediates the survival disparity between those with and without dementia, highlighting the importance of patient‐centered discussions that balance the risks and benefits of surgical treatment.

## 1 Introduction

Colorectal cancer (CRC) is the second leading cause of cancer‐related death in the United States, with a median age at diagnosis of 66 years and an average age at death of 72 years [1]. Dementia affects an estimated 57.4 million people worldwide, and its prevalence is projected to increase to 152.8 million by 2050 [2]. Advancing age is a major risk factor for both CRC and dementia; as the US population continues to age, the number of patients living with both CRC and dementia is expected to rise.

Multiple studies have shown that a preexisting diagnosis of dementia is associated with higher mortality among patients with cancer [3, 4, 5, 6, 7]. This association is thought to reflect, in part, older age, more advanced stage at presentation, and differences in cancer management [8]. Patients with dementia and colon cancer undergo invasive diagnostic evaluation less often, receive chemotherapy and surgery at lower rates, and are more likely to receive no cancer‐directed treatment [5]. No clinical guidelines specifically address cancer treatment in patients with concomitant dementia. Consequently, care often deviates from standard guideline‐concordant pathways, informed by shared decision‐making with patients and families and by clinician assessment of the risks and benefits of invasive and/or potentially debilitating treatments [9]. These differences in treatment may contribute to observed survival disparities. For example, in a study by Chen et al., reduced survival among individuals with CRC and dementia was 13% mediated by a lower likelihood of receiving adjuvant chemotherapy [10].

Although many individuals with CRC undergo curative‐intent oncologic resection [11, 12, 13], and timely surgery is associated with improved survival [14], the extent to which differences in surgical management between patients with and without dementia contribute to survival disparities remains unknown. Further understanding of this relationship could help inform treatment plans and discussions of informed consent with patients and families. The objectives of this study were to (1) compare demographics and treatment patterns among individuals with CRC with versus without dementia in our state, and (2) evaluate the causal relationship between dementia, receipt or refusal of surgery for Stage I–III colon cancer, and survival. We hypothesized that differences in surgical treatment practices partially mediate the association between dementia and increased mortality.

## 2 Methods

We conducted a retrospective, population‐based cohort study using the Indiana State Cancer Registry, a statewide registry with routine linkage to state and national vital records. We identified adults (≥ 18 years) with CRC diagnosed between 2000 and 2020, defined by registry site codes for cancers of the colon or rectum. Noninvasive tumors, or no staging information and cases with a missing diagnosis date or vital status were excluded.

All eligible CRC cases were included in descriptive and survival analyses. Patients with missing information on surgery or key covariates, including vital status, were excluded. Patients with all other data but with non‐positive follow‐up time were excluded from the mediation cohort.

The primary exposure was the presence of dementia, defined by the presence of International Classification of Diseases, Ninth or Tenth Revision (ICD‐9/10) (see Table S1) codes for dementia in registry comorbidity or secondary diagnosis fields. Patients with at least one such code were classified as having dementia; all others were classified as not having dementia.

We summarized baseline characteristics and treatment patterns by dementia status and compared groups using standard statistical methods. Overall survival was estimated using Kaplan–Meier methods and compared between patients with and without dementia using log‐rank tests, both in the full CRC cohort and in a subgroup of patients aged ≥ 80 years.

The primary outcome was overall survival, defined as time from cancer diagnosis to death from any cause, with patients alive at last contact censored at that date. Vital status and follow‐up dates were obtained from the registry.

The primary mediator was receipt of cancer‐directed colon surgery as part of the first course of treatment, as recorded in registry treatment fields. We also used structured “reason for no surgery” codes to identify documented refusal of surgery, which was examined descriptively and in exploratory mediation analyses. Refusal was only documented in cases where surgery was offered and recommended but declined by the patient. Use of chemotherapy and radiation therapy was ascertained to characterize treatment patterns.

Covariates were selected a priori as potential confounders of the relationships among dementia, treatment, and survival, including age at diagnosis, sex, race/ethnicity, number of comorbidities (excluding dementia), primary payer, anatomic subsite (colon vs. other colorectal sites), and a simplified summary stage category. These analyses were repeated in a subgroup of patients over the age of 80 to specifically examine a population with a shorter survival horizon due to age‐related factors independent of dementia, and to assess whether the presence of dementia further influenced treatment decisions and survival within this high‐risk group.

To assess the extent to which the association between dementia and survival was mediated by surgery utilization and refusal, we conducted regression‐based causal mediation analyses within a counterfactual framework for time‐to‐event outcomes. Mediation analysis was used to assess the extent to which the relationship between the exposure (dementia) and the outcome (survival) operates through an intermediate variable, or mediator, in this case, receipt of surgery. We jointly modeled receipt of surgery using logistic regression and overall survival using a parametric accelerated failure time model with a Weibull distribution, adjusting for the covariates listed above. Under standard causal mediation assumptions, we estimated total percent mediated, natural direct, and natural indirect effects of dementia on survival, expressed as mean survival time ratios, with confidence intervals obtained from bootstrap resampling [15]. The direct effect is the difference in survival between patients with and without dementia after making their likelihood of receiving surgery the same as it would be for comparable patients without dementia. The indirect effect is the part of the survival difference that is specifically due to dementia changing who receives surgery, and the survival consequences of that change. This was repeated with surgery refusal as a mediator within the same cohort but assessing differing refusal rates in those with and without dementia (see Figure S1). Missing covariate data were addressed using multiple imputations by chained equations, which creates multiple completed datasets by iteratively imputing each incomplete variable from a model conditional on the others. The mediation models were then fit in each imputed dataset, and the results were combined across imputations. Observations with missing information in variables not imputed (or otherwise required for model fitting) were omitted as needed by the regression procedures. All analyses were performed using R (R version 4.5.2, Vienna, Austria).

## 3 Results

In the overall cohort of patients meeting the inclusion criteria, there were 69,318; of these, 1516 (2.1%) had dementia and 67,802 (97.8%) did not (Figure 1). There was a higher percentage of female patients in the dementia cohort (55%) compared to patients without dementia (49%) (*p* < 0.001). Both groups were predominantly white (91% in both cohorts), and the dementia cohort had a higher percentage of black patients (8.3% vs. 7.7%; *p* = 0.13). Patients in the dementia cohort were also older, with a median age at diagnosis of 79 (vs. 68, *p* < 0.001). A higher percentage of patients in the dementia cohort also had Medicare (83% vs. 55%, *p* < 0.001). Regarding cancer characteristics, there were significant differences in cancer location between cohorts. In the dementia cohort, 84% of cancers were in the colon, and 16% in the rectum. Conversely, the non‐dementia cohort had a lower percentage of colon cancer at 78%, with higher percentages of rectal cancers, 22% (*p* < 0.001).

**FIGURE 1:**
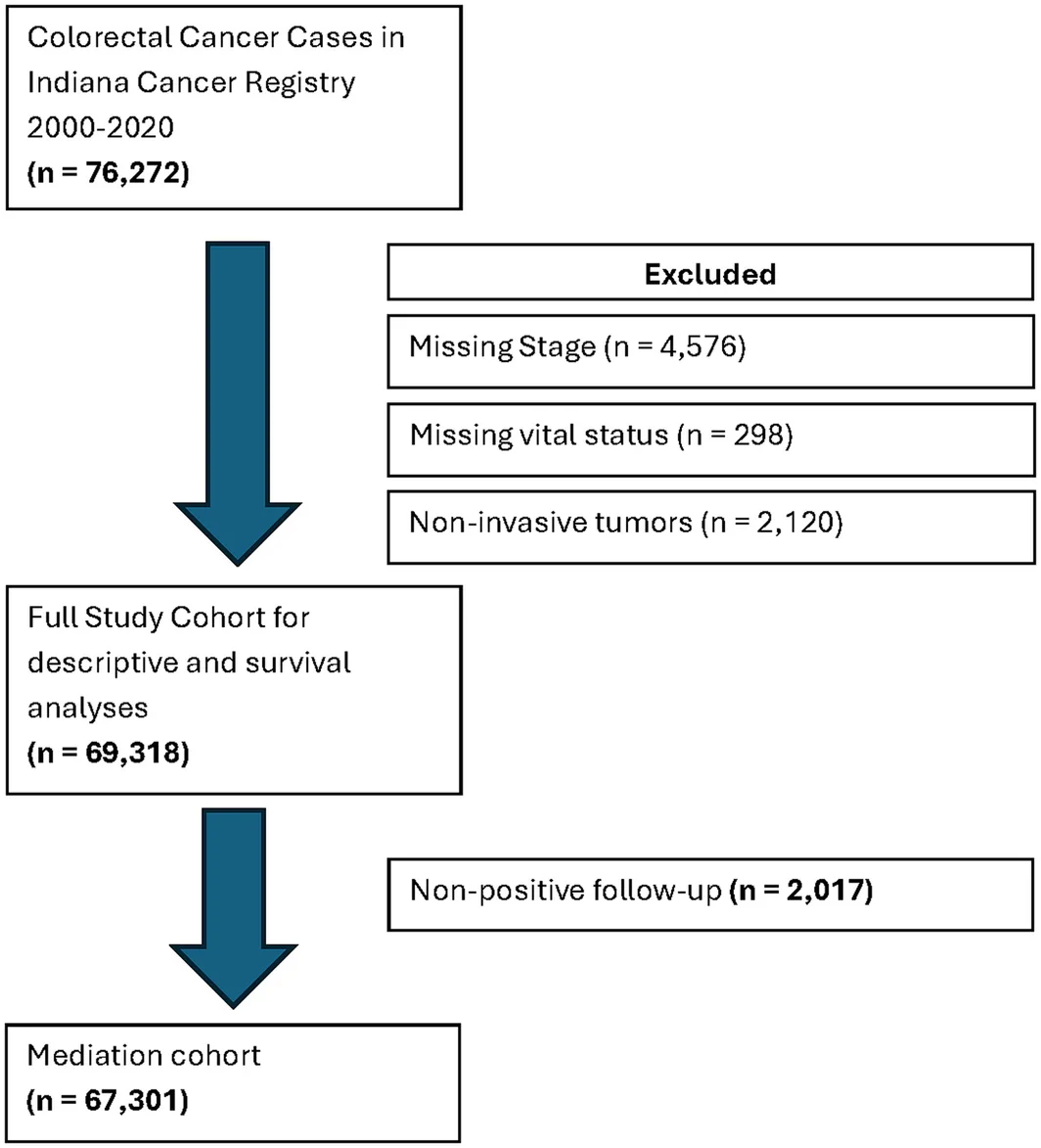
Patient attrition diagram.

Regarding differences in management between groups, overall management differed significantly (*p* < 0.001). Patients with dementia had higher rates of no treatment (20% vs. 12%) and surgery‐only (62% vs. 59%) treatment compared to patients without dementia, all of these findings were significant with *p* < 0.001. Patients without dementia had higher rates of neoadjuvant therapy with surgery (1.4% vs. 1.1%), chemotherapy with or without radiation (4.9% vs. 3.2%), and surgery with adjuvant chemotherapy (18% vs. 10%). Patients with dementia had higher rates of not receiving recommended surgery (3.7% vs. 1.6%) and radiation (1.9% vs. 1.0%) due to deemed contraindicating patient factors which are not explicitly specified within the database but may include comorbidities, including dementia, as well as cancer or other treatment considerations. Patients in the dementia cohort also specifically had significantly higher rates of refusal of recommended surgery (4.1% vs. 1.3%, *p* < 0.001) and radiation therapy (1.9% vs. 0.9%, *p* < 0.001). A full breakdown of patient demographics, cancer characteristics, and management is provided in Table 1.

**TABLE 1:** Patient characteristics and treatment of patients with colorectal cancer by dementia status.

Characteristics	*N*	No dementia	Dementia	*p* ^b^
		*N* = 67,802^a^	*N* = 1516^a^	
Race	69,318			0.13
Asian		283 (0.4%)	4 (0.3%)	
Black		5213 (7.7%)	126 (8.3%)	
Other/unknown		339 (0.5%)	2 (0.1%)	
White		61,967 (91.3%)	1384 (91.3%)	
Sex: Female	69,318	33,207 (48.9%)	840 (55.4%)	< 0.001
Age at diagnosis	69,318			< 0.001
Median (Q1, Q3)		69 (59, 78)	79 (71, 86)	
Chemotherapy	69,318	17,218 (25.4%)	227 (14.9%)	< 0.001
Radiation	68,081			< 0.001
No		58,028 (85.6%)	1385 (91.4%)	
Yes		8546 (12.6%)	122 (8.1%)	
Received surgery	69,318	55,747 (82.2%)	1141 (75.2%)	< 0.001
SEER staging	69,316			< 0.001
Distant		13,026 (19.2%)	288 (19.0%)	
Localized		28,117 (41.4%)	623 (41.1%)	
Regional, lymph node		23,403 (34.5%)	490 (32.3%)	
Unknown/unstaged		3254 (4.8%)	115 (7.6%)	
Cancer site	69,318			< 0.001
Colon (excludes appendix)		53,056 (78.3%)	1266 (83.5%)	
Rectum		14,746 (21.7%)	250 (16.5%)	
Treatment regimen	67,323			< 0.001
Neoadjuvant + surg		947 (1.4%)	16 (1.1%)	
No treatment		7590 (11.2%)	295 (19.5%)	
Only chemo +/− rad		3197 (4.9%)	48 (3.2%)	
Other/unknown		4242 (6.3%)	43 (2.8%)	
Rad only		1016 (1.5%)	30 (2.0%)	
Surg + chemo		12,083 (17.8%)	153 (10.1%)	
Surg only		38,727 (57.1%)	931 (61.4%)	
Reason for no surgery	69,318			
Surgery was performed		55,747 (82.2%)	1141 (75.2%)	
Not part of the planned first course treatment		7303 (10.7%)	217 (14.3%)	
Contraindicated due to patient factors		1060 (1.6%)	56 (3.7%)	
Patient died prior to surgery		144 (0.2%)	6 (0.4%)	
Was recommended but not performed, NOS		691 (1.0%)	1 (< 0.1%)	
Recommended but refused by patient, family, or guardian		893 (1.3%)	62 (4.1%)	
Unknown if performed		421 (0.6%)	10 (0.7%)	
Unknown if recommended or performed		1543 (2.3%)	23 (1.5%)	
Insurance status	69,318			< 0.001
Medicaid		2333 (3.4%)	37 (2.4%)	
Medicare		37,428 (55%)	1263 (83%)	
Other/unknown		13,065 (19.2%)	72 (4.7%)	
Private		13,354 (19.7%)	127 (8.4%)	
Uninsured		1622 (2.4%)	17 (1.1%)	
Unknown		6579 (9.7%)	10 (0.7%)	
Surgery refusal	69,318	893 (1.3%)	62 (4.1%)	< 0.001
Radiation refusal	68,081	612 (0.9%)	28 (1.9%)	< 0.001

^a^ *n* (%).

^b^ Pearson's Chi‐squared test; Fisher's exact test; Wilcoxon rank sum test; NA.

When assessing individuals over the age of 80 with colon cancer, there were 15,885 individuals meeting criteria, 734 (4.6%) with dementia, and 15,151 (95.4%) without dementia. Both groups had higher percentages of female patients, 65% and 60%, respectively. There was no difference in race between groups. The dementia group was, on average, older, at 86 years versus 84 years (*p* < 0.001). There was no significant difference in tumor location between groups. Individuals in this subgroup without dementia had higher rates of chemotherapy (10% vs. 5.6%; *p* < 0.001), radiation (6.3% vs. 4.4%; *p* = 0.04), and surgery (75% vs. 69%; *p* < 0.001). They also had a higher rate of surgery refusal (6.4% vs. 3.5%, *p* < 0.001), but no difference in radiation refusal (Table 2).

**TABLE 2:** Patient characteristics and treatment of patients with colorectal cancer by dementia status (age 80+).

Characteristics	*N*	No dementia	Dementia	*p* ^b^
		*N* = 15,151^a^	*N* = 734^a^	
Sex: Female	15,885	9111 (60.0%)	479 (65.3%)	0.006
Age at diagnosis	15,885			< 0.001
Median (Q1, Q3)		84.0 (82.0, 88.0)	86.0 (83.0, 89.0)	
Race	15,885			0.2
Asian		27 (0.2%)	1 (0.1%)	
Black		815 (5.4%)	51 (6.9%)	
Other/unknown		23 (0.2%)	0 (0%)	
White		14,286 (94.4%)	682 (92.9%)	
Chemotherapy	15,885	1555 (10.3%)	41 (5.6%)	< 0.001
Radiation	15,531	933 (6.3%)	32 (4.4%)	0.036
Received surgery	15,885	11,310 (74.7%)	504 (68.7%)	< 0.001
SEER staging	15,885			0.042
Distant		2478 (16.4%)	105 (14.3%)	
Localized		6142 (40.5%)	296 (40.3%)	
Regional, lymph node		5134 (33.9%)	244 (33.2%)	
Unknown/unstaged		1397 (9.2%)	89 (12.1%)	
Cancer site	15,885			0.4
Colon (excludes appendix)		12,860 (84.9%)	631 (86.0%)	
Rectum		2291 (15.1%)	103 (14.0%)	
Reason for no surgery	15,885			
Surgery was performed		11,310 (74.7%)	504 (68.7%)	
Not part of the planned first course treatment		2012 (13.3%)	122 (16.6%)	
Contraindicated due to patient factors		469 (3.1%)	37 (5.0%)	
Patient died prior to surgery		39 (0.3%)	5 (0.7%)	
Was recommended but not performed, NOS		242 (1.6%)	1 (0.1%)	
Recommended but refused by patient, family, or guardian		529 (3.5%)	47 (6.4%)	
Unknown if performed		63 (0.4%)	4 (0.5%)	
Unknown if recommended or performed		487 (3.2%)	14 (1.9%)	
Refused surgery	15,885	529 (3.5%)	47 (6.4%)	< 0.001
Radiation refusal	15,531	268 (1.8%)	17 (2.3%)	0.3
Insurance status	15,885			
Medicaid		60 (0.4%)	6 (0.8%)	
Medicare		12,785 (84.4%)	706 (96.2%)	
Other/unknown		1961 (12.9%)	14 (1.9%)	
Private		264 (1.7%)	6 (0.8%)	
Uninsured		81 (0.5%)	2 (0.3%)	

^a^ *n* (%).

^b^ Pearson's Chi‐squared test; Wilcoxon rank sum test; Fisher's exact test; NA.

In subsequent survival analyses, patients without dementia at any age had higher survival probabilities across all time points (12, 36, 60, 120, and 180 months), and the survival difference was statistically significant (*p* < 0.001). In individuals over the age of 80, patients without dementia also had higher survival probabilities across all time points, which was significant (*p* < 0.001) (shown in Figure 2).

**FIGURE 2:**
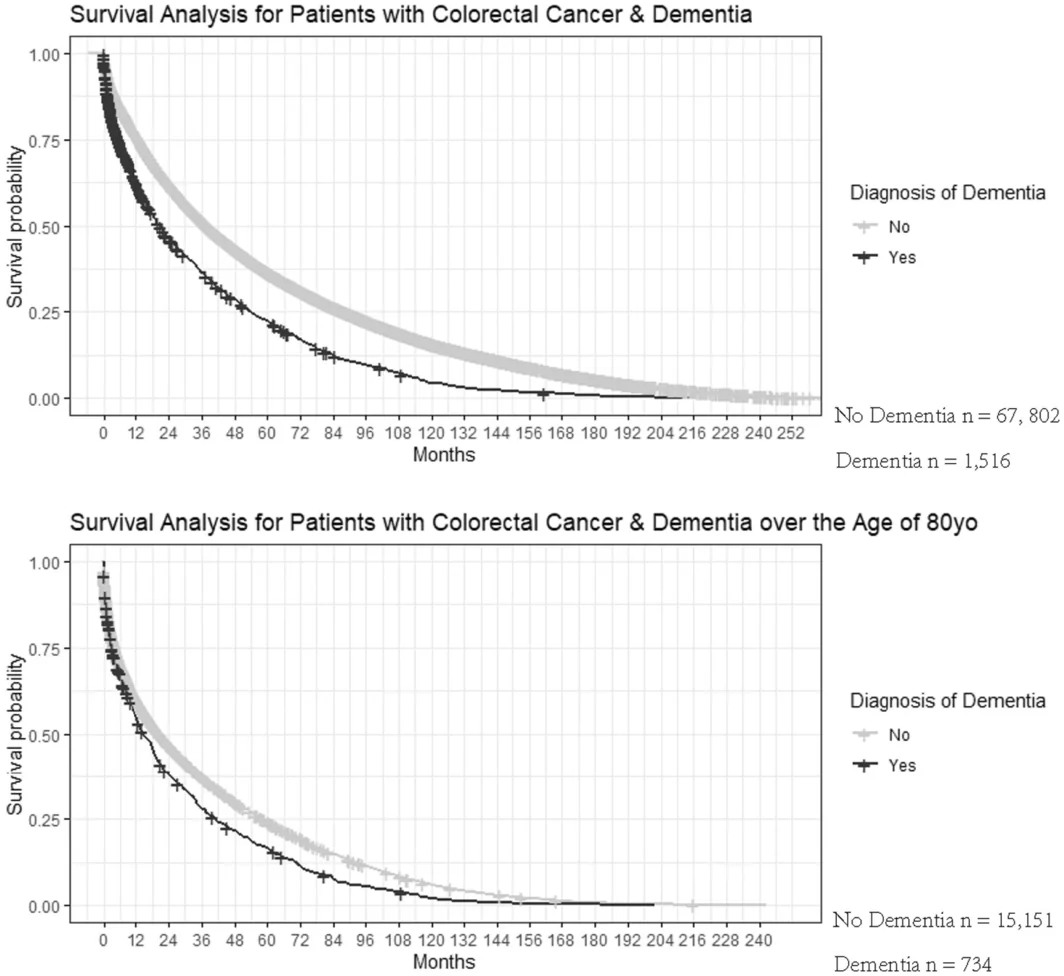
Survival curves from time of diagnosis for patients with colorectal cancer and dementia.

In the mediation analysis, the natural direct effect (time ratio 0.78, 95% CI 0.7–0.8) indicated a 22.1% decrease in mean survival time for individuals with dementia and CRC compared with those without dementia under a counterfactual scenario in which surgery receipt was set to the level expected if dementia were absent. The natural indirect effect (time ratio = 0.98; 95% CI, 0.96–0.99) indicated an additional 2% reduction in mean survival time attributable to dementia‐related differences in surgical receipt. Overall, surgery was a statistically significant mediator of the association between dementia and survival, with a proportion mediated of 9.6% (95% CI 6.2%–13.2%) (Table 3). Regarding the mediating effect of surgery refusal rates, the natural direct effect was 0.78 (95% CI 0.7–0.8), and the natural indirect effect was 0.99 (95% CI 0.995–0.999). Overall, surgery refusal was a statistically significant mediator, with a mediated proportion of 0.95% (95% CI: 0.2%–1.7%) (Table 3).

**TABLE 3:** Mediation analyses of surgery receipt and surgery refusal on dementia‐related survival disparities.

Mediation analysis: Effect of surgery receipt on dementia‐related survival disparities		
Effect	Estimate	Interpretation
Total effect (TTE)	0.78	22.3% reduction in mean survival
Natural direct effect (NDE)	0.78	Direct effect (not via surgery)
Natural indirect effect (NIE)	0.98	Indirect effect (via surgery)
Proportion mediated	9.6%	Surgery explains 9.6% of the survival gap

Mediation analysis: Effect of surgery refusal on dementia‐related survival disparities		
Effect	Estimate	Interpretation
Total effect (TTE)	0.78	22.3% reduction in mean survival
Natural direct effect (NDE)	0.78	Direct effect (not via surgery refusal)
Natural indirect effect (NIE)	0.997	Indirect effect (via surgery refusal)
Proportion mediated	0.95%	Surgery refusal explains 0.95% of the survival gap

## 4 Discussion

Individuals with dementia and cancer experience greater variability in treatment and worse overall survival than individuals without dementia [3, 4, 5, 7, 8, 10, 16]. In our study, patients with dementia were less likely to receive chemotherapy, radiation, or surgery, and they had higher rates of refusal of surgery and radiation; overall survival was lower in the overall cohort and among patients aged 80 years and older. We also found that differences in surgical utilization partially accounted for the decreased survival observed among individuals with dementia and colon cancer.

We found that individuals with dementia and CRC experienced differences in management, including lower rates of chemotherapy, radiation, and surgery, consistent with prior studies. These differences in surgical utilization and refusal likely reflect shared decision‐making among clinicians, patients, and families that incorporates goals of care and quality‐of‐life considerations [9, 17]. In addition, dementia has been reported as an independent risk factor for postoperative complications and mortality, which may further influence surgical decision‐making [18, 19, 20, 21, 22]. This risk likely reflects both the physiologic stress of major surgery and the ways cognitive impairment can limit participation in postoperative care, rehabilitation, and recovery. Together, these factors may shape discussions between clinicians and families and contribute to lower surgical recommendations and higher refusal rates.

We also found in our state cohort, consistent with prior studies [3, 4, 7, 10] Individuals with dementia and CRC had lower overall survival than those without dementia. Notably, survival was lower at every evaluated postoperative interval, from 30 through 180 days.

Surgery has long been a mainstay of CRC treatment, and most patients, including those in the geriatric population, undergo curative‐intent oncologic resection [11, 12]. However, treatment decisions for patients with dementia are often complex, requiring clinicians, patients, and families to balance the risks of the cancer against the risks of surgery in the context of baseline quality of life, functional status, and life expectancy. These considerations may help explain the treatment variation observed in prior studies and in our cohort.

Importantly, dementia exists on a spectrum, and severity has substantial implications for both quality of life and survival. In a study of 4033 patients with dementia and CRC, 2‐year survival declined markedly with increasing dementia severity, ranging from 53.5% among those with mild dementia to 16.5% among those with severe dementia [6]. Against this backdrop, our mediation analysis adds evidence that differences in surgical practice contribute to survival disparities, demonstrating that variation in surgical utilization significantly mediated the association between dementia and decreased survival, with a mediating effect of 9.6%. This means that approximately 10% of the overall survival disparity of those with CRC and dementia can be attributed to the difference in surgical utilization, which is similar in magnitude to the 13% mediating effect found by Chen et al. relating to omission of chemotherapy [10]. Although our findings indicate a mediating effect of surgery refusal on survival of only 0.95%, the actual receipt of surgery is likely a more reliable and important consideration. We speculate that refusal may be underrepresented in this database, as a substantial proportion of patients had surgery recorded as ‘not part of the first course of treatment’ despite surgery being the guideline‐recommended approach for most Stage I–III cases. In some instances, goals‐of‐care discussions may have led physicians to forgo recommending surgery based on anticipated patient preferences, which would not be captured as refusal by data abstractors unless explicitly documented as such in the medical record.

These findings do not diminish the importance of patient‐centered shared decision‐making, or the fact that the decision of providers to not offer, or patients to not move forward with surgery is appropriate in many cases. Rather, they can inform surgical discussions with patients and families by clarifying that foregoing surgery may carry measurable survival consequences for some patients. Although some decisions to avoid surgery will appropriately align with goals of care, our results also suggest there may be a subset of individuals with dementia who could benefit from surgical management at rates more comparable to peers without dementia.

This study has several limitations. First, the cancer registry database did not include information about CRC staging for all patients, and therefore we may have underestimated the true differences in staging between groups. However, our simplified staging variable did capture key features that generally preclude curative‐intent surgery, such as distant metastasis. The registry did not include information relevant to clinical outcomes, including functional status and support, frailty, and goals of care including code status at the time of cancer diagnosis. Second, it is well documented that younger patients with CRC often exhibit distinct tumor characteristics, biology, and treatment responsiveness, and this may have introduced a confounder in the survival comparisons; true early‐onset CRC comprised only a small proportion of our cohort. Controlling for age at diagnosis within the mediation analysis helps account for this variability to some extent, though it cannot be fully controlled for. Dementia status was identified using diagnostic codes, and data on dementia subtype or severity were unavailable, precluding evaluation of whether treatment patterns and outcomes differed across the dementia spectrum which may have led to under ascertainment of dementia cases and not including mild cognitive impairment prior to definitive diagnosis. The timing of dementia diagnosis was not specified so this may alter the causal pathway of some patients diagnosed after cancer diagnosis but this would still be relevant to survival. The use of diagnosis codes in databases has variable reliability in identifying dementia. However, this has been shown to have low sensitivity, but it is highly specific, indicating that the vast majority of cases included truly had dementia [23, 24]. Finally, although a reason for nonreceipt of surgery was documented, most cases were classified as “not part of the primary treatment plan” without additional detail, or “not recommended due to patient factors,” limiting insight into the underlying rationale, including goals‐of‐care discussions, patient and family preferences, or other clinical considerations informing decision‐making. Therefore, it is possible that actual refusal may be underestimated.

To our knowledge, this study is the first to specifically evaluate how variation in surgical management among individuals with dementia and CRC affects survival. We found that patients with dementia were less likely to undergo surgery, even after adjustment for relevant clinical factors, and that these differences in surgical practice were associated with decreased survival, findings that should be considered during treatment planning. Accordingly, high‐quality care for this population requires thorough, patient‐centered discussions among patients, clinicians, and families that address goals of care, baseline quality of life, and the likely benefits and burdens of surgical treatment.

## 5 Conclusion

We found that individuals with CRC and dementia received lower rates of chemotherapy, radiation, and surgery, had higher rates of refusal of recommended surgery and radiation, and experienced decreased survival following cancer diagnosis. Differences in surgical management between individuals with CRC with and without dementia significantly mediated the observed survival disparity. These findings underscore the importance of comprehensive, patient‐centered discussions among patients, families, and care teams regarding goals of care, baseline quality of life, and the anticipated benefits and burdens of surgical treatment. While surgery may not be appropriate or aligned with the goals of many patients, these discussions must acknowledge that a subset of patients may derive a survival benefit from surgical intervention.

## Author Contributions

**E.M.A.:** primary writing, and revision of manuscript and data handling. **V.V.M.:** conceptualization of project and feedback and revisions of manuscript. **M.E.L.:** conceptualization, data handling, and analysis. **B.R.:** data analysis and handling. **M.A.B.:** project conceptualization and feedback and revisions of manuscript. **S.M.:** primary investigator, project conceptualization, and feedback and revisions of manuscript.

## Funding

This work was supported by Melvin and Bren Simon Cancer Center, Indiana University (5K23AG071945). E.M.A. is supported on a training grant from the National Cancer Institute (NCI T32CA282070).

## Disclosure

The funding sources had no role in the design, conduct, or analysis of the study, or in the preparation or review of the manuscript.

## Conflicts of Interest

The authors declare no conflicts of interest.

## Supporting information


**Table S1:** ICD 9/10 codes used for identification of dementia.
**Figure S1:** Directed acyclic graph (DAG) modeling relationship with dementia and surgery receipt and refusal and mortality.
**Figure S1:** The yellow node represents the exposure (dementia), the blue nodes represent the outcome (mortality) and mediators of interest (surgery, surgery refusal), and white nodes represent covariates included as potential confounders (age, sex, comorbidities, cancer stage, cancer site, and insurance status). Green arrows indicate causal paths along the exposure‐outcome relationship, and black arrows indicate other assumed causal relationships in the model.
