# Supplementary material for: Surgery Utilization Mediates Survival in Patients With Dementia and Stage I–III Colorectal Cancer
Source: J Am Geriatr Soc. 2026 Aug 7;74(10):2997–3005. doi: 10.1111/jgs.70643 (PMC13723200; doi:10.1111/jgs.70643)
Supplement: Supplementary file 1 [file JGS-74-2997-s001.pdf]

Table S1: ICD 9/10 Codes Used for Identification of Dementia

290, 291.2, 292.82, 294, 331, F00, F01, F02, F03, G20, F05.1, G31.1, I69.91, R41

Figure S1. Directed Acyclic Graph (DAG) Modeling Relationship with Dementia and Surgery Receipt and Refusal and Mortality

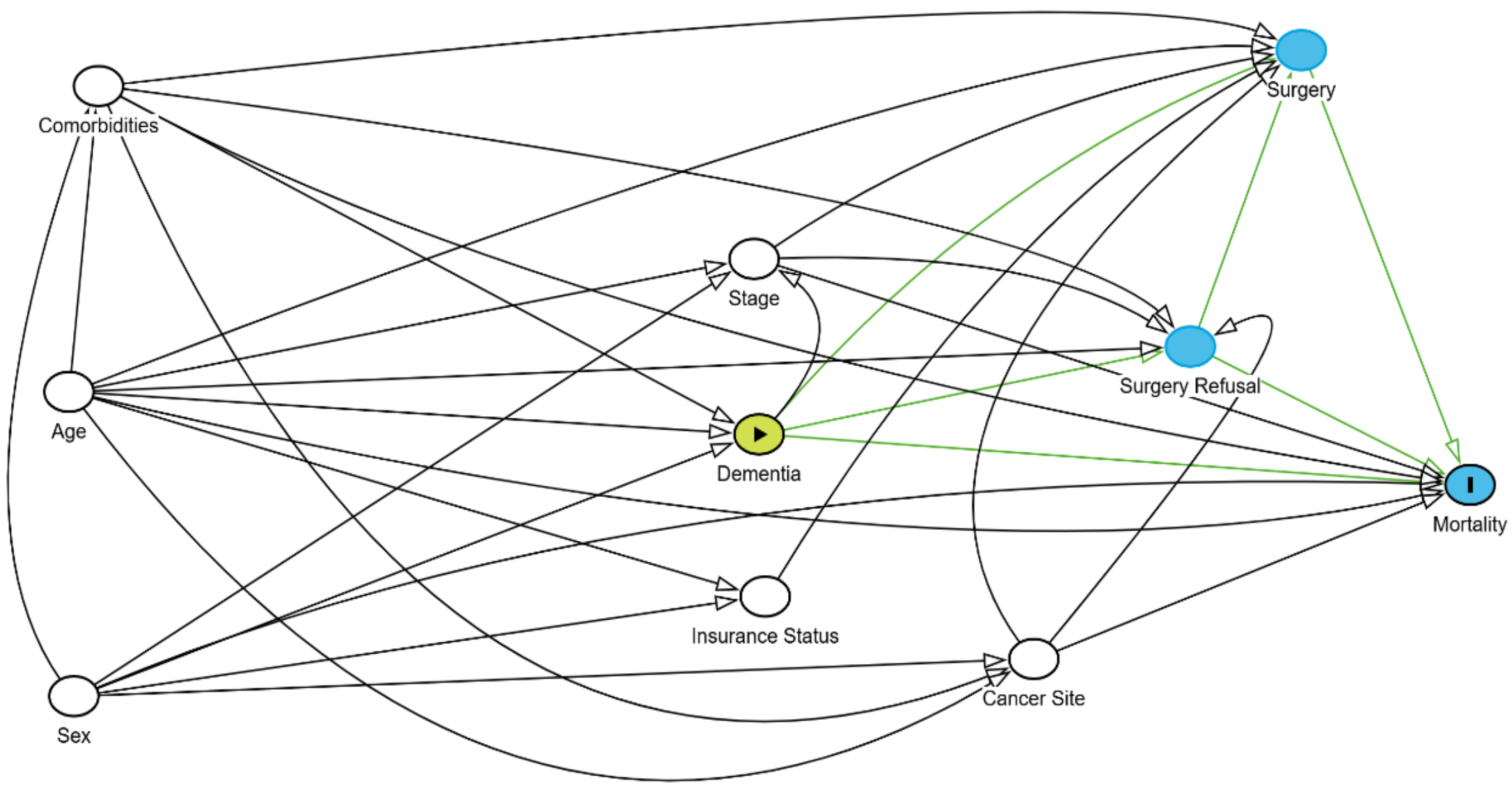

The yellow node represents the exposure (dementia), the blue nodes represent the outcome (mortality) and mediators of interest (surgery, surgery refusal), and white nodes represent covariates included as potential confounders (age, sex, comorbidities, cancer stage, cancer site, and insurance status). Green arrows indicate causal paths along the exposure-outcome relationship, and black arrows indicate other assumed causal relationships in the model.
